# Species-Specific, pH-Independent, Standard Redox Potential of Selenocysteine and Selenocysteamine

**DOI:** 10.3390/antiox9060465

**Published:** 2020-06-01

**Authors:** Tamás Pálla, Arash Mirzahosseini, Béla Noszál

**Affiliations:** 1 Department of Pharmaceutical Chemistry, Semmelweis University, H-1092 Budapest, Hungary; palla.tamas@pharma.semmelweis-univ.hu (T.P.); mirzahosseini.arash@pharma.semmelweis-univ.hu (A.M.); 2 Research Group of Drugs of Abuse and Doping Agents, Hungarian Academy of Sciences, H-1051 Budapest, Hungary

**Keywords:** selenocysteine, redox, disulfide

## Abstract

Microscopic redox equilibrium constants and standard redox potential values were determined to quantify selenolate-diselenide equilibria of biological significance. The highly composite, codependent acid-base and redox equilibria of selenolates could so far be converted into pH-dependent, apparent parameters (equilibrium constants, redox potentials) only. In this work, the selenolate-diselenide redox equilibria of selenocysteamine and selenocysteine against dithiothreitol were analyzed by quantitative nuclear magnetic resonance (NMR) methods to characterize the interfering acid-base and redox equilibria. The directly obtained, pH-dependent, conditional redox equilibrium constants were then decomposed by our method into pH-independent, microscopic constants, which characterize the two-electron redox transitions of selenocysteamine and selenocysteine. The 12 different, species-specific parameter values show close correlation with the respective selenolate basicities, providing a tool to estimate otherwise inaccessible site-specific selenolate-diselenide redox potentials of related moieties in large peptides and proteins.

## 1. Introduction

The role of selenium, the biological trace element and the related selenium-containing proteins (selenoproteins) has been described in numerous antioxidant processes [1]. Selenocysteine, known as the twenty-first amino acid, is the only selenium-containing building block of proteins that contains selenium. The majority of selenoproteins are enzymes (such as glutathione peroxidase [2], iodothyronine deiodinase [3], thioredoxin reductase [4]), most of which are involved in redox reactions. Their selenocysteine residue is the essential unit for their catalytic activity.

A new, specific way to quantify redox properties at the submolecular level has recently been introduced to characterize thiols of biological importance (cysteine, cystamine, homocysteine and glutathione [5,6]; ovothiol A and penicillamine [5,7]). A major conclusion of these studies is that there is a close correlation between the thiolate basicities and the thiolate-disulfide redox properties [5,6,7,8,9]. The hypothesis of this work is that such a correlation between acid-base and redox characteristics exists for the selenium-analogues of the above compounds as well, namely for selenocysteine and selenocysteamine. We wish to demonstrate said correlation by determining the species-specific physico-chemical parameters of the acid-base and redox equilibria of the above compounds and extend the correlation to nuclear magnetic resonance (NMR) chemical shift values as well.

**Figure 1:**
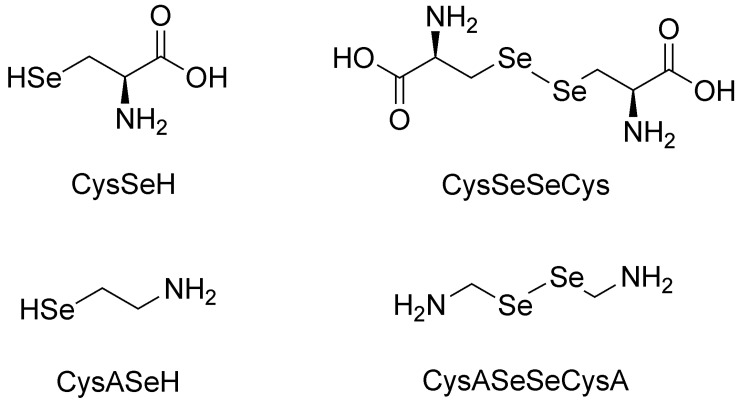
The structure of the investigated selenols (left) and diselenides (right).

In this work, the species-specific redox equilibria of two biogenic selenols: selenocysteamine (CysASeH) and selenocysteine (CysSeH) with dithiothreitol (DTT) were studied. The oxidation of these compounds yields selenocystamine (CysASeSeCysA) and selenocystine (CysSeSeCys), respectively. The constitutional formulae of the studied selenium-containing compounds are depicted in Figure 1.

Glutathione is known as a “gold standard” in thiol-disulfide biochemistry, hence, every thiol-containing antioxidant is compared to glutathione. Moreover, the species-specific physico-chemical acid-base [9] and redox [5,6] parameters of glutathione are well-characterized. However, previous works examining selenoproteins or selenium-containing peptides found that the diselenide bridge cannot be reduced by glutathione [10,11]. We found that even a large excess of glutathione was ineffective in reducing the studied diselenide compounds, confirming the above observation. Thus, a more powerful reducing agent was needed to investigate selenolate-diselenide redox equilibria. Based on literature recommendations [8,12], dithiothreitol (DTT), a stronger reducing agent, was chosen. Dithiothreitol undergoes a one-step two-electron redox reaction, resulting in the formation of oxidized dithiothreitol (DTT_ox_) with an intramolecular disulfide in a six-membered ring. During the redox processes between dithiothreitol and other agents, the presence of the intermediate compound, where only one of the thiolate groups of DTT^2−^ is oxidized is negligible [13]. The main physico-chemical properties of DTT are already described (log*K*_1_ = 10.1; log*K*_2_ = 9.2 [14]; *E*° = −0.323 V at pH = 7.0 [15]).

**Figure 2:**
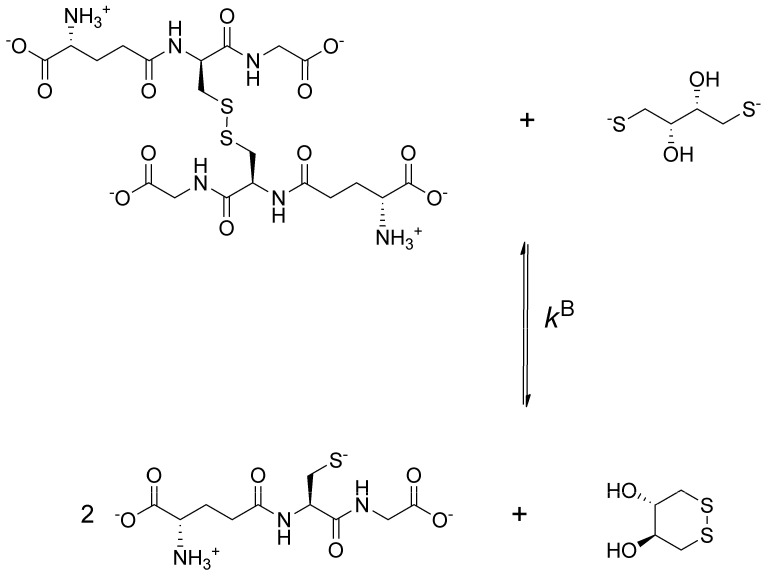
The redox reaction between oxidized glutathione and dithiothreitol (DTT)^2−^ dominant microspecies at the pH of blood.

To obtain data describing selenolate-diselenide redox equilibria comparable to those of thiolate-disulfide systems, first the glutathione-DTT^2−^ redox reaction was investigated (Figure 2).

**Figure 3:**
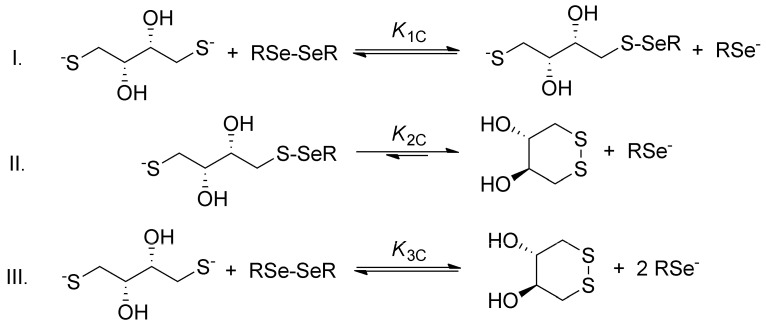
The two-step redox equilibria between a diselenide compound and DTT^2−^ (reaction I and II) followed by their aggregated one-step net reaction (III). Identical reaction schemes are applicable for disulfide analogues. The arrow in reaction II indicates the low probability of formation of the sulfur–selenium bridge.

The general step-by-step scheme of selenolate-diselenide redox equilibria with DTT^2−^ is depicted in Figure 3, where RSe^−^ and RSeSeR stand for the reduced and oxidized form of the selenium-containing compounds, respectively. The reaction between disulfides (e.g., oxidized glutathione) and DTT^2−^ is analogous (where RS^−^ and RSSR stand for the thiolate and disulfide, respectively).

Since DTT^2−^ is highly driven towards the ring-closing second oxidation step [13], and diselenides are more stable than the selenylsulfides [16], the reaction between diselenides and DTT^2−^ can be reasonably discussed in terms of the net reaction. The conditional equilibrium constant describing this net reaction is as follows:

(1) $$K_{3C}=\frac{{\left[{\mathrm{RSe}}^{-}\right]}^{2}\left[{\mathrm{DTT}}_{\mathrm{ox}.}\right]}{\left[{\mathrm{DTT}}^{2-}\right]\left[\mathrm{RSeSeR}\right]}$$

**Figure 4:**
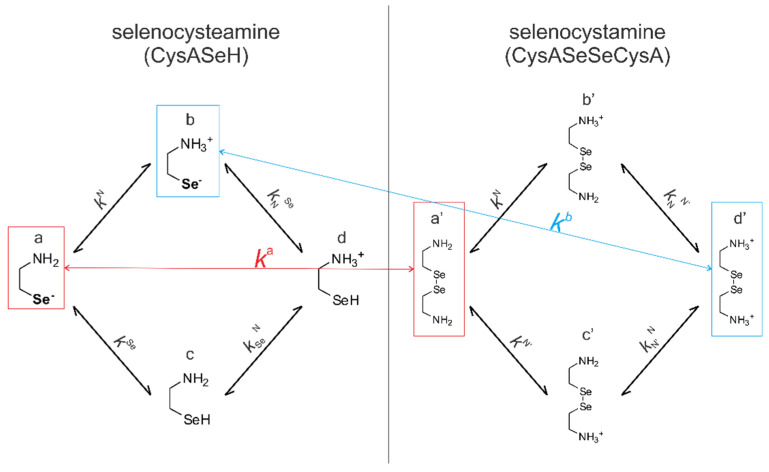
The coexisting protonation and redox microequilibria of selenocysteamine and selenocystamine; N, and Se labels denote the amino, and selenolate groups, respectively.

**Figure 5:**
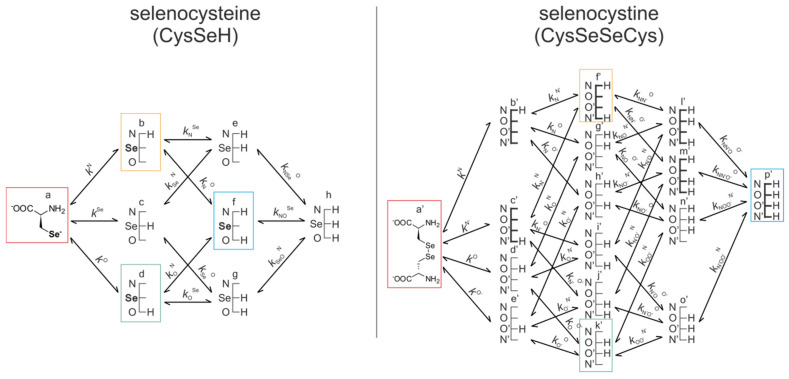
The protonation microequilibrium schemes of selenocysteine (left) and selenocystine (right). For simplicity, not every protonation microspecies is shown in a structural formula, rather a schematic structure depicts the microspecies with its basic sites where N, Se, and O labels denote the amino, selenolate, and carboxylate groups, respectively. The corresponding species participating in redox equilibria are bordered in color.

Since redox and acid-base reactions coexist, the observed apparent redox equilibrium constants are pH-dependent. In order to get a clear insight into the redox equilibria, purified from the protonation effects, an improved evaluation method has been introduced [5,6]. This new method makes it possible to determine species-specific redox equilibrium constants (*k*^x^) and the species-specific, i.e., the standard redox potential (*E*°_x_, where x is the general sign of the appropriate RSe^−^ microspecies in Figure 4 and Figure 5). This determination method requires all the species-specific protonation constants, including those of the minor microspecies; otherwise, redox processes could only be characterized at the level of phenomenon. The complete set of the microscopic protonation constants of the studied selenium-containing compounds has recently been established [17]. The protonation constants of selenocysteine, selenocysteamine, and glutathione, relevant to this work, are collected in Table 1 and used for further calculations.

Here, we report the comprehensive redox characterization of the selenolate-diselenide equilibria of selenocysteine and selenocysteamine in terms of pH-independent standard redox potentials (298 K, 95/5 *v*/*v*% H_2_O/D_2_O and 0.15 mol/L ionic strength). The correlation between the acid-base and redox parameters with NMR chemical shifts is also determined.

## 2. Methods

### 2.1. Materials

Seleno-l-cystine, oxidized glutathione, sarcosine and *tert*-butylamine were obtained from Sigma Aldrich, dithiothreitol was obtained from Tokyo Chemical Industry, selenocystamine dihydrochloride was purchased from AKos GmbH. Deuterium oxide (D_2_O) and methanol were purchased from Merck. All reagents were of analytical grade and used without further purification. The deionized water was prepared with a Milli-Q Direct 8 Millipore system.

### 2.2. Preparation of Solutions for Equilibrium Constant Determination

Stock solutions containing one of the reactants (seleno-l-cystine, selenocystamine dihydrochloride, oxidized glutathione or dithiothreitol) were prepared by dissolving in borax buffer. To a stock solution of DTT^2−^ a known amount of *tert*-butylamine or sarcosine was added for use as an internal concentration standard and in situ pH indicator (see NMR spectroscopy chapter). A series of solutions was made by mixing the two types of stock solutions followed by pH adjustment using hydrochloric acid or sodium hydroxide [19]. The NMR measurement was carried out immediately after preparing the sample solution to diminish the risk of oxidation by air. To ensure that equilibrium was reached, NMR spectra were recorded instantly, 15 min, 1 h and 24 h after preparation of the sample. As no difference was detected between the spectra, the onset of the equilibrium is considered immediate, which is in good agreement with the kinetics of thiolate-disulfide transitions [20]. A blank series (with no selenol compound) was also prepared and measured to determine the perturbing effect of oxidation by air.

### 2.3. NMR Spectroscopy

NMR spectra were recorded on a Varian 600 MHz spectrometer at 298 K. The solvent in every case was H_2_O:D_2_O, 95:5, *v*/*v* (0.15 mol/L ionic strength), using internal DSS (3-(trimethylsilyl)propane-1-sulfonate sodium) as chemical shift reference. The sample volume was 600 μL, the analyte concentration was 1–5 mmol/L. The pH values were determined by internal indicator molecules optimized for NMR [21,22]. The water resonance was diminished by presaturation pulse sequence (nt = 16, np = 64,000, acquisition time = 301 ms, relaxation delay = 15 s).

### 2.4. Data Analysis

For the analysis of quantitative NMR measurements the Lorentzian peak fitting algorithm of the ACD/NMR Processor Academic Edition v12.01 software package (Advanced Chemistry Development, Toronto, ON, Canada) was used with automatic baseline and phase correction, and no apodization. The integrals of the fitted peaks were compared to the integral of the concentration standard peak for concentration determination. For the regression analyses, the software Origin Pro 8 (OriginLab Corp., Northampton, MA, USA) was used.

## 3. Results

Figure 4 shows the species-specific protonation equilibria of selenocysteamine and selenocystamine, along with the selenolate → diselenide oxidation processes. Microspecies that take part in the redox processes are framed.

Figure 5 represents the species-specific acid-base equilibria of selenocysteine and selenocystine. The corresponding microspecies in the redox pairs have identical status of the amino and carboxylate sites.

Acid-base equilibria are often described with macroscopic protonation constants, which only reveal the stoichiometry of the successively protonated ligands. Microscopic protonation schemes used in this work, however, characterize the site of protonation as well. The protonation microspecies are discerned with their one-letter symbols (a, b, c, etc.) and the microscopic protonation constants are depicted using *k*^N^, *k*^Se^, *k*^O^, etc. The superscript of microscopic protonation constants *k* indicates the protonating group, while the subscript (if any) shows the site(s) already protonated. Se, N, O denote the selenolate, amino, and carboxylate sites, respectively.

The acid-base microequilibria are indispensable constituents in the evaluation of the species-specific, pH-independent redox equilibrium constants, as shown in Equations (7) and (8). Some protonation constant examples for selenocysteine are shown below:

(2) $$K_{1}=\frac{\left[{\mathrm{HL}}^{-}\right]}{[L^{2-}]\left[H^{+}\right]}$$

(3) $$\beta_{3}=K_{1}K_{2}K_{3}=\frac{\left[H_{3}L^{+}\right]}{[L^{2-}]{\left[H^{+}\right]}^{3}}$$

(4) $$k^{N}=\frac{\left[b\right]}{\left[a\right]\left[H^{+}\right]}$$

where *K*_1_, *K*_2_, *K*_3_ are successive macroconstants, *β*_3_ is one of the cumulative macroconstants, and *k*^N^ is the microconstant representing the amino protonation in CysSeH, when its carboxylate and selenolate sites are unprotonated. The concentrations of the various macrospecies comprise the sum of the concentration of those microspecies that contain the same number of protons, for example, in CysSeSeCys:

(5) $$\left[H_{2}L\right]=\left[\;f^{\prime }\right]+\left[\;g^{\prime }\right]+\left[\;h^{\prime }\right]+\left[\;i^{\prime }\right]+\left[\;j^{\prime }\right]+\left[\;k^{\prime }\right]$$

For the selenolate-diselenide (and dithiolate-disulfide for dithiothreitol) redox equilibria, only the apparent (pH-dependent) or conditional equilibrium constants (*K*_3C_) are directly available, by determining the equilibrium concentration of the compounds (RSeH, DTT, RSeSeR, and DTT_ox._) which correspond to the total concentration of variously protonated reactant species. The pH-dependent, apparent redox equilibrium constants can be decomposed into pH-independent, species-specific equilibrium constants, the number of which is large, but definite. For example, the selenolate moiety in CysSeH is adjacent to two other basic sites: the amino and the carboxylate. Therefore, the number of selenolate-bearing microspecies is four, corresponding to the number of protonation states of the amino and carboxylate moieties (amino-carboxylate, ammonium-carboxylate, amino-carboxyl. ammonium-carboxyl). Accordingly, the selenolate exists in four different electrostatic environments, having thus four different oxidizabilities. Considering the opposite direction of the redox equilibrium, dithiothreitol has only one microspecies, bearing both thiols in their deprotonated, oxidizable form. Therefore, the redox equilibria between selenocysteine and dithiothreitol can be characterized with four different microscopic, species-specific equilibrium constants. The method for determining the microscopic redox equilibrium constants is analogous for every case. The determination of one of the microscopic redox equilibrium constants, *k*^b^ will be demonstrated. The notation *k*^b^ refers to the reaction involving the ”b” microspecies of CysSeH and oxidized dithiothreitol on the products side, and the corresponding ”f′“ microspecies of CysSeSeCys and the deprotonated dithiothreitol on the reactants side. Note that ”b” defines that the diselenide microspecies is mandatorily ”f′”, since ”f′“ is the only diselenide microspecies with protonation states in the side-chain identical with ”b”. Superscript ”b” therefore unambiguously identifies all four microspecies in the microequilibrium in question. The example of this pH-independent, microscopic selenolate-diselenide equilibrium constant is in Equation (6):

(6) $$k^{b}=\frac{{\left[b\right]}^{2}\left[{\mathrm{DTT}}_{\mathrm{ox}.}\right]}{\left[{\mathrm{DTT}}^{2-}\right]\left[f^{\prime }\right]}$$

For the next step of the calculation, the relative abundance of the protonation microspecies is needed. The mole fraction of microspecies ”b” relative to the total CysSeH concentration can be written with the following equation, as can be carried out for every microspecies provided the complete set of protonation microconstants is known.

(7) $$\left[b\right]=\left[\mathrm{CysSeH}\right]\chi_{b}=\left[\mathrm{CysSeH}\right]\frac{k^{N}\left[H^{+}\right]}{1+K_{1}\left[H^{+}\right]+K_{1}K_{2}{\left[H^{+}\right]}^{2}+K_{1}K_{2}K_{3}{\left[H^{+}\right]}^{3}}$$

By writing analogous equations as Equation (7) for the remaining microspecies in the formula of *k*^b^, one can write the equation of the microscopic equilibrium constant expressed with the conditional equilibrium constant as follows:

(8) $$k^{b}=\frac{{\left[b\right]}^{2}\left[{\mathrm{DTT}}_{\mathrm{ox}.}\right]}{\left[{\mathrm{DTT}}^{2-}\right]\left[f^{\prime }\right]}=\frac{{\left[\mathrm{CysSeH}\right]}^{2}\cdot \chi_{b}^{2}\cdot \left[{\mathrm{DTT}}_{\mathrm{ox}.}\right]}{\left[\mathrm{DTT}\right]\cdot \chi_{{\mathrm{DTT}}^{2-}}\cdot \left[{\mathrm{Se}}_{2}\mathrm{Cys}\right]\cdot \chi_{f^{\prime }}}=K_{3C}\frac{\chi_{b}^{2}}{\chi_{{\mathrm{DTT}}^{2-}}\cdot \chi_{f^{\prime }}}$$

Thus, if *K*_3C_ and the *χ* values are known, the species-specific redox microconstants can be calculated. The conditional redox equilibrium constant values determined directly from the NMR spectra are listed in Table 2 and Supplementary Materials. These conditional redox equilibrium constants were determined in basic media only (above pH 9) due to the fact that the redox reaction did not commence at lower pH values. Only the negatively charged thiolate in DTT^2−^ participates in redox reactions, and below pH 9 the thiolate groups are overwhelmingly in protonated form. However, as the conditional redox equilibrium constants provide pH-independent redox microconstants, it is apparent from Equation (8) that the pH range of determination does not impede the calculation. It should be noted however, that the pH-independent redox microconstants pertaining to microspecies that are predominantly present at pH values outside the determined range (i.e., *k*^f^) will have a greater inherent uncertainty based on the present calculations.

The microscopic redox equilibrium constants were calculated and derived from the conditional equilibrium constants using analogous equations to Equation (8) based on at least three repeated measurements. The mean values of the calculated microscopic redox equilibrium constants and their standard deviation of determination are listed in Table 3. Note that near physiological pH (i.e., around pH 7) the most abundant CysSe and CysASe selenolate microspecies are ”b” and ”b”, respectively. In fact, these microspecies have maximal relative abundance between pH 6.5 and 10, therefore the pH range of determination in our study is well-conditioned for the extraction of the physiologically relevant redox microconstants.

**Figure 6:**
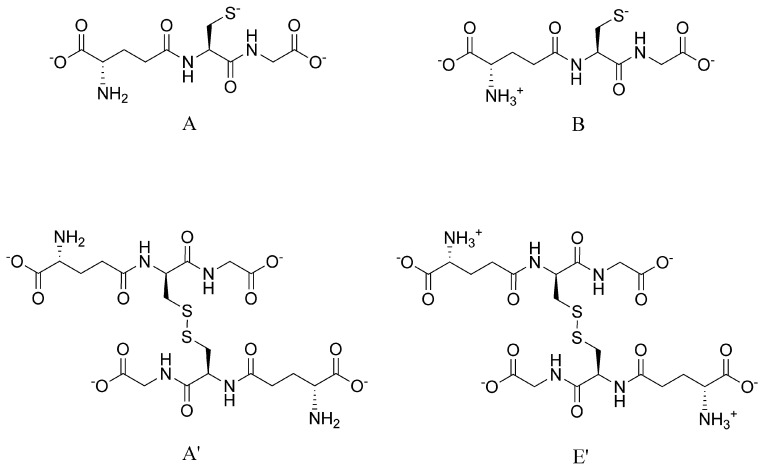
The structure and protonation states of the referenced glutathione (A, B, top) and glutathione disulfide (A’, E’, bottom) microspecies in this work.

In order to obtain the standard redox potential values of the studied selenol compounds, the standard redox potential of dithiothreitol is needed. Although dithiothreitol has been extensively studied, only its apparent redox potential has been previously determined, therefore we determined the pH-independent, species-specific standard redox potential of DTT_ox._/DTT^2−^ as well using glutathione as the reaction partner. In the state of a chemical equilibrium, the electrode potential of every existing redox system is equal. For example, for the reaction of dithiothreitol and glutathione one can write:

(9) $$E_{{\mathrm{DTT}}_{\mathrm{ox}.}/{\mathrm{DTT}}^{2-}}=E_{\;A^{\prime }/A}=E_{\;E^{\prime }/B}=\cdots$$

where the A’/A, E’/B symbols denote the microspecies of glutathione disulfide/glutathione redox pairs. We chose to perform further calculations with equations pertaining to glutathione/glutathione disulfide microspecies B/E’, since this redox pair has the highest relative abundance in the pH of interest, and therefore its calculation is least exposed to uncertainty. The above equation adapted for glutathione microspecies ”B” Figure 6 and DTT^2−^ becomes:

(10) $$E{{}^{\circ}}_{{\mathrm{DTT}}_{\mathrm{ox}.}/{\mathrm{DTT}}^{2-}}+\frac{RT}{zF}\ln\frac{\left[{\mathrm{DTT}}_{\mathrm{ox}.}\right]}{\left[{\mathrm{DTT}}^{2-}\right]}=E{{}^{\circ}}_{\;E^{\prime }/B}+\frac{RT}{zF}\ln\frac{\left[\;E^{\prime }\right]}{{\left[B\right]}^{2}}$$

(11) $$E{{}^{\circ}}_{{\mathrm{DTT}}_{\mathrm{ox}.}/{\mathrm{DTT}}^{2-}}=E{{}^{\circ}}_{\;E^{\prime }/B}+\frac{RT}{zF}\ln\frac{\left[\;E^{\prime }\right][{\mathrm{DTT}}^{2-}]}{{\left[B\right]}^{2}\left[{\mathrm{DTT}}_{\mathrm{ox}.}\right]}=E{{}^{\circ}}_{\;E^{\prime }/B}-\frac{RT}{zF}\ln k^{B}$$

where *E*° is the standard redox potential of the redox system denoted in its subscript, *R* is the universal gas constant, *T* is the absolute temperature, *z* is the number of electrons transferred in the redox half-reaction, and *F* is the Faraday constant. Using the previously determined microscopic protonation constant [9] and standard redox potential [5] of glutathione microspecies ”B”, the species-specific standard redox potential was determined for the DTT_ox._/DTT^2−^ redox system as −0.403 ± 0.007 V.

With the knowledge of the selenolate-diselenide species-specific redox equilibrium constants and the standard redox potential of the DTT_ox._/DTT^2−^ redox couple, the species-specific standard redox potentials of the different selenolate-containing microspecies could be calculated. Equation (12) shows the continued example of CysSeH microspecies ”b”. The comprehensive set of determined species-specific standard redox potentials is listed in Table 4.

(12) $$E{{}^{\circ}}_{b}=E{{}^{\circ}}_{{\mathrm{DTT}}_{\mathrm{ox}.}/{\mathrm{DTT}}^{2-}}+\frac{RT}{zF}\ln\frac{{\left[b\right]}^{2}\left[{\mathrm{DTT}}_{\mathrm{ox}.}\right]}{\left[\;f^{\prime }\right][{\mathrm{DTT}}^{2-}]}=E{{}^{\circ}}_{{\mathrm{DTT}}_{\mathrm{ox}.}/{\mathrm{DTT}}^{2-}}+\frac{RT}{zF}\ln k^{b}$$

**Figure 7:**
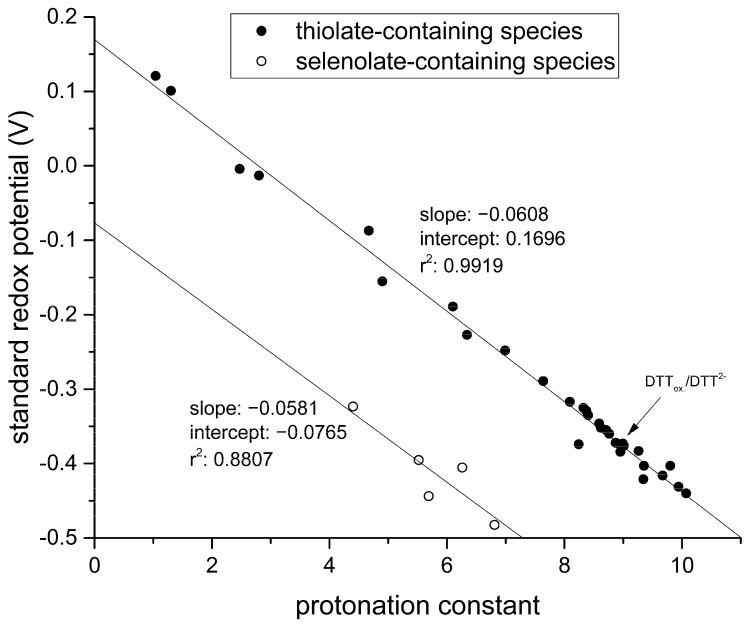
Correlation between standard redox potentials and species-specific thiolate protonation constants for various thiol-containing compounds (glutathione, cysteine, cysteamine, homocysteine, penicillamine, ovothiol A) reproduced from [4] with the addition of the data of dithiothreitol (upper line, full circles); the correlation between standard redox potentials and the species-specific selenolate protonation constants for the various selenol-containing compounds (lower line, empty circles).

By graphing these standard redox potentials against the concomitant selenolate-specific protonation constants compiled in Table 1 we find a similar correlation to that of thiolates presented in Figure 7.

## 4. Discussion

In this work, the highly interwoven acid-base and redox pathways of selenolate-diselenide systems were decomposed into species-specific, component equilibria. The standard redox potentials of biologically relevant selenolate-diselenide couples are determined for the first time; these values characterize the redox processes at the protonation microspecies level. The elucidation of these redox microequilibria reveals considerable differences between the various protonation species. The knowledge of species-specific standard redox potentials of selenocysteine and selenocystine, in particular, improves our knowledge on redox homeostasis and can lead to better interpretation of several biochemical phenomena. For example the cytoprotective potential of selenoproteins is thought to entail several mechanisms involving thio/seleno chemistry [23]. One proposed mechanism of action is formation of stable diselenide bonds in thioredoxin reductase acting as a ”diselenide trap”, which can be further supported by exact standard redox potentials of the reaction partners. New selenoproteins are investigated for their function, likely related to catalyzing thiol/disulfide exchange in proteins, as the kinetics of selenolate-diselenide transitions is seven orders of magnitude greater than that of thiolate-disulfide transitions [24]. In order to understand these catalytic effects purified from the protonation fraction of the thiolate and selenolate moieties at physiological pH, a comprehensive species-specific characterization is needed. The knowledge of detailed physico-chemical properties of selenocompounds presented in this work can also serve as the basis to develop artificial selenoenzymes [25] of highly selective redox capacities.

The example of selenocystine microspecies ”a′” and ”f′” (Table 4) shows that even side chain protonation changes can lead to significantly different redox characteristics, with nearly 160 mV difference in standard redox potential values. Therefore, small changes in pH can not only affect the redox processes of selenolate-diselenide transitions by changing the protonation fraction of the selenolate, but also by altering the protonation state of neighboring moieties.

**Figure 8:**
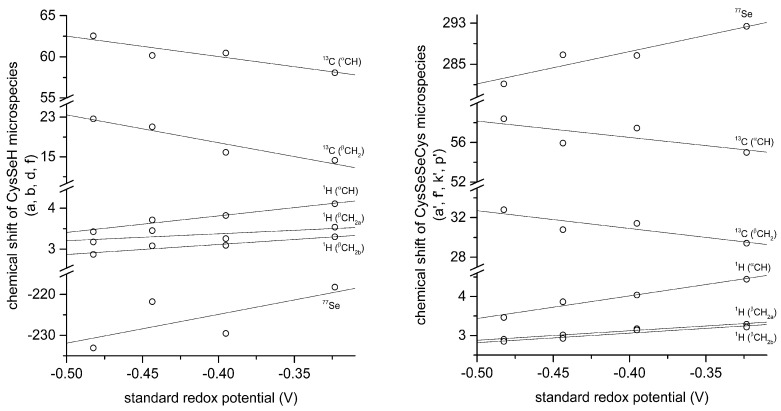
Correlation of selenocysteine standard redox potentials with the corresponding selenocysteine microspecies chemical shifts (left) and selenocystine microspecies chemical shifts (right).

The correlation between selenolate basicity and standard redox potentials verifies the previous observations regarding thiolate basicity and its proportionality with thiolate oxidizability. It is interesting that the correlation line between selenolate basicity and the concomitant standard redox potential is parallel to the correlation line of thiolates, however shifted by ca. −246 mV units. This accentuates the fact that selenolates, apart from being less basic, are vastly stronger reducing agents than thiolates in general. It is noteworthy that the species-specific standard redox potentials of selenolate-containing microspecies also show linear correlation with species-specific NMR chemical shift. The chemical shift values were previously determined [16], and the details of the correlation are shown in Figure 8 and Table 5. The use of chemical shifts in protein NMR (from ^1^H, ^13^C, and for selenoproteins the relatively undisrupted ^77^Se spectra) can now serve as sound means to predict selenolate oxidizability or diselenide reducibilty/stability in proteins: a key parameter to understand and influence oxidative stress.

The critical issue in designing preventive or therapeutic antioxidants is the narrow path of redox potentials, effective enough to reduce harmful oxidative agents, but keeps disulfides and other reducible units in useful biomolecules intact. Naturally, small reducing agents can hardly be as selective as substrate-specific enzymes of the biological antioxidant system; however, a finely tuned and designed selenolate-containing compound with an appropriate basicity, redox potential and concomitant selectivity can be confined to a narrower range. The correlation between the redox and NMR parameters serves now as a sound basis to better quantify the characteristics of diselenide moieties in selenoenzymes, allowing thus the development of potent, selective antioxidant compounds for serious ailments related to selenoenzyme deficiencies, such as autism [26].

## 5. Conclusions

The standard redox potentials of diselenide/selenolate-containing microspecies of selenocysteine and selenocysteamine were determined using an indirect approach of measuring redox equilibrium constants with the help of dithiothreitol. These standard redox potentials are pH-independent and show correlation with selenolate acid-base characteristics; an important observation previously demonstrated for thiolate-analogues as well. The acid-base and redox parameters both show correlation with the NMR chemical shifts of the diselenide or selenolate-containing species as well. This can be used as a tool for predicting diselenide behavior solely based on ^77^Se NMR data.

## Figures and Tables

**Table 1:** Protonation constant and standard redox potential values of the studied compounds from previous works. See Figure 4 and Figure 5 for the description of the equilibrium constants.

Selenocysteine [17]		Selenocystine [17]		Selenocysteamine [17]		Glutathione [9]		Oxidized Glutathione [18]	
log*K*_1_	10.18	log*K*_1_	9.19	log*K*_1_	10.87	log*K*_1_	9.65	log*K*_1_	9.53
log*K*_2_	5.52	log*K*_2_	8.22	log*K*_2_	6.26	log*K*_2_	8.78	log*K*_2_	8.83
log*K*_3_	2.01	log*K*_3_	2.25	log*k*^N^	10.87	log*K*_3_	3.52	log*K*_3_	3.85
log*k*^N^	10.18	log*K*_4_	1.58	log*k*^Se^	7.55	log*K*_4_	2.22	log*K*_4_	3.15
log*k*^O^	5.02	log*k*^N^	8.89	log*k*_N_^Se^	6.26	logk^N^	9.49	log*K*_5_	2.32
log*k*_N_^O^	3.13	log*k*_N_^N’^	8.52			*E*°_B_	−0.374 mV	log*K*_6_	1.60
log*k*^Se^	6.81	log*k*^O^	4.40	Selenocystamine [17]		Dithiothreitol [14]		log*k*^N^	9.41
log*k*_N_^Se^	5.52	log*k*_O_^O’^	4.33	log*K*_1_	9.62	log*K*_1_	10.1	log*k*_N_^N’^	9.25
log*k*_O_^Se^	5.69			log*K*_2_	8.48	log*K*_2_	9.2		
log*k*_NO_^Se^	4.40								

**Table 2:** The conditional redox equilibrium constants in logarithmic units at the pH indicated in reaction mixtures of dithiothreitol with selenocysteine, selenocysteamine and glutathione.

Selenocysteine		Selenocysteamine		Glutathione	
pH	log*K*_C_	pH	log*K*_C_	pH	log*K*_C_
9.79	−2.88	9.66	−2.31	9.00	0.00
9.81	−2.20	9.67	−2.43	8.30	−0.41
9.86	−2.87	9.67	−2.23	8.35	−0.03
9.90	−2.07	9.68	−2.48	9.07	0.20
9.96	−1.98	9.69	−2.40	9.63	0.41
9.98	−2.04	9.80	−2.37	10.23	0.05
10.02	−2.05	9.89	−2.09	11.01	0.83
		9.94	−2.25		
		9.98	−2.21		
		10.01	−2.16		

**Table 3:** Microspecies-specific, pH-independent equilibrium constants of selenocysteine, selenocysteamine and glutathione with dithiothreitol in logarithmic units, with standard deviations. The parameter of glutathione was determined for the B microspecies only, for further calculation (see Figure 6 for glutathione microspecies).

Selenocysteine			Selenocysteamine			Glutathione		
Microspecies	log*k*	*sd*	Microspecies	log*k*	*sd*	Microspecies	log*k*	*sd*
a	−2.68	0.42	a	−3.72	0.19	B	0.99	0.24
b	0.27	0.42	b	−0.08	0.19			
d	−1.37	0.42						
f	2.70	0.42						

**Table 4:** The species-specific standard redox potential values for every microspecies of selenocysteine and selenocysteamine given as mean ± standard deviation.

Selenocysteine		Selenocysteamine	
Microspecies	*E*° (V)	Microspecies	*E*° (V)
a	−0.482 ± 0.01	a	−0.513 ± 0.006
b	−0.395 ± 0.01	b	−0.405 ± 0.006
d	−0.444 ± 0.01		
f	−0.323 ± 0.01		

**Table 5:** Correlation data of selenocysteine standard redox potentials with the corresponding selenocysteine microspecies chemical shifts and the corresponding selenocystine microspecies chemical shifts.

Selenocysteine						
	^77^Se	^1^H (^α^CH)	^1^H (^β^CH_2a_)	^1^H (^β^CH_2b_)	^13^C (^α^CH)	^13^C (^β^CH_2_)
slope	70.524	5.251	2.208	3.163	−24.743	−52.487
intercept	−196.660	5.755	3.970	4.013	50.137	−3.319
*r* ^2^	0.4979	0.9622	0.4838	0.9095	0.8571	0.9180
**Selenocystine**						
slope	59.106	5.806	2.446	2.437	−16.557	−18.030
intercept	310.960	6.338	4.102	4.036	49.893	23.692
*r* ^2^	0.8908	0.9660	0.9720	0.9259	0.5578	0.7718

## Supplementary Materials

The following are available online at https://www.mdpi.com/2076-3921/9/6/465/s1.

## References

[1] Tapiero H., Townsend D.M., Tew K.D. (2003). The antioxidant role of selenium and seleno-compounds. Biomed. Pharmacother..

[2] Flohe L., Günzler W., Schock H. (1973). Glutathione peroxidase: A selenoenzyme. FEBS Lett..

[3] Köhrle J. (2000). The deiodinase family: Selenoenzymes regulating thyroid hormone availability and action. Cell. Mol. Life Sci. CMLS.

[4] Zhong L., Arnér E.S., Holmgren A. (2000). Structure and mechanism of mammalian thioredoxin reductase: The active site is a redox-active selenolthiol/selenenylsulfide formed from the conserved cysteine-selenocysteine sequence. Proc. Natl. Acad. Sci. USA.

[5] Mirzahosseini A., Noszál B. (2016). Species-specific standard redox potential of thiol-disulfide systems: A key parameter to develop agents against oxidative stress. Sci. Rep..

[6] Mirzahosseini A., Somlyay M.T., Noszál B.L. (2015). Species-specific thiol-disulfide equilibrium constant: A tool to characterize redox transitions of biological importance. J. Phys. Chem. B.

[7] Mirzahosseini A., Noszál B. (2016). Species-specific thiol-disulfide equilibrium constants of ovothiol A and penicillamine with glutathione. RSC Adv..

[8] Keire D.A., Strauss E., Guo W., Noszal B., Rabenstein D.L. (1992). Kinetics and equilibria of thiol/disulfide interchange reactions of selected biological thiols and related molecules with oxidized glutathione. J. Org. Chem..

[9] Mirzahosseini A., Somlyay M., Noszál B. (2015). The comprehensive acid-base characterization of glutathione. Chem. Phys. Lett..

[10] Koide T., Itoh H., Otaka A., Yasui H., Kuroda M., Esaki N., Soda K., Fujii N. (1993). Synthetic Study on Selenocystine-Contaning Peptides. Chem. Pharm. Bull..

[11] Singh R., Whitesides G.M. (1991). Selenols catalyze the interchange reactions of dithiols and disulfides in water. J. Org. Chem..

[12] Guenther W.H. (1967). Methods in selenium chemistry. III. Reduction of diselenides with dithiothreitol. J. Org. Chem..

[13] Cleland W.W. (1964). Dithiothreitol, a new protective reagent for SH groups. Biochemistry.

[14] Whitesides G.M., Lilburn J.E., Szajewski R.P. (1977). Rates of thiol-disulfide interchange reactions between mono-and dithiols and Ellman’s reagent. J. Org. Chem..

[15] Szajewski R.P., Whitesides G.M. (1980). Rate constants and equilibrium constants for thiol-disulfide interchange reactions involving oxidized glutathione. J. Am. Chem. Soc..

[16] Besse D., Budisa N., Karnbrock W., Minks C., Musiol H.-J., Pegoraro S., Siedler F., Weyher E., Moroder L. (1997). Chalcogen-analogs of amino acids. Their use in X-ray crystallographic and folding studies of peptides and proteins. Biol. Chem..

[17] Pálla T., Mirzahosseini A., Noszál B. (2020). The species-specific acid-base and multinuclear magnetic resonance properties of selenocysteamine, selenocysteine, and their homodiselenides. Chem. Phys. Lett..

[18] Noszál B., Szakács Z. (2003). Microscopic protonation equilibria of oxidized glutathione. J. Phys. Chem. B.

[19] Bates R.G., Bower V.E. (1956). Alkaline solutions for pH control. Anal. Chem..

[20] Mirzahosseini A., Faragó Z., Noszál B. (2018). Determination of pH-independent rate constants of thiolate-disulfide redox transitions. New J. Chem..

[21] Orgován G., Noszál B. (2011). Electrodeless, accurate pH determination in highly basic media using a new set of 1 H NMR pH indicators. J. Pharm. Biomed. Anal..

[22] Szakács Z., Hägele G., Tyka R. (2004). 1H/31P NMR pH indicator series to eliminate the glass electrode in NMR spectroscopic pKa determinations. Anal. Chim. Acta.

[23] Ganther H.E. (1999). Selenium metabolism, selenoproteins and mechanisms of cancer prevention: Complexities with thioredoxin reductase. Carcinogenesis.

[24] Pleasants J.C., Guo W., Rabenstein D.L. (1989). A comparative study of the kinetics of selenol/diselenide and thiol/disulfide exchange reactions. J. Am. Chem. Soc..

[25] Huang X., Liu X., Luo Q., Liu J., Shen J. (2011). Artificial selenoenzymes: Designed and redesigned. Chem. Soc. Rev..

[26] Raymond L.J., Deth R.C., Ralston N.V. (2014). Potential role of selenoenzymes and antioxidant metabolism in relation to autism etiology and pathology. Autism Res. Treat..

