# Supplementary material for: Species-Specific, pH-Independent, Standard Redox Potential of Selenocysteine and Selenocysteamine
Source: Antioxidants (Basel). 2020 Jun 1;9(6):465. doi: 10.3390/antiox9060465 (PMC7346207; doi:10.3390/antiox9060465)
Supplement: Supplementary file 1 [file antioxidants-09-00465-s001.pdf]

Supplementary Material

Species-Specific, pH-Independent, Standard Redox Potential of Selenocysteine and Selenocysteamine

Tamás Pálla [1,2], Arash Mirzahosseini [1,2] and Béla Noszál [1,2,*]

[1] Department of Pharmaceutical Chemistry, Semmelweis University, Budapest H-1092, Hungary; palla.tamas@pharma.semmelweis-univ.hu (T.P.); mirzahosseini.arash@pharma.semmelweis-univ.hu (A.M.)

[2] Research Group of Drugs of Abuse and Doping Agents, Hungarian Academy of Sciences, Budapest H-1051, Hungary

* Correspondence: noszal.bela@pharma.semmelweis-univ.hu; Tel.: +36-1217-0891

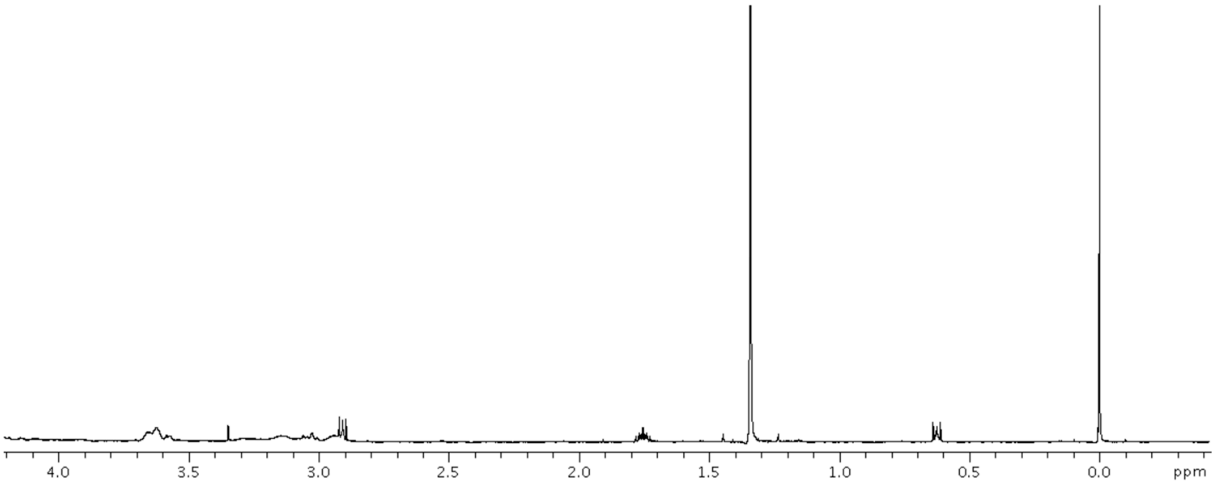

Figure S1. ^{1}H NMR spectrum of reaction mixture at equilibrium of selenocystine with dithiothreitol at pH 9.79.

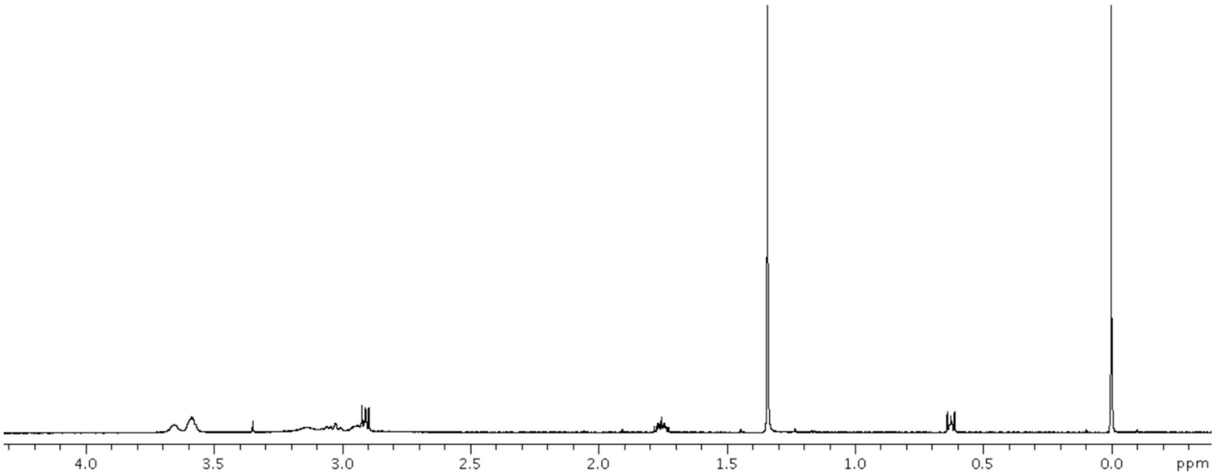

Figure S2. ^{1}H NMR spectrum of reaction mixture at equilibrium of selenocystine with dithiothreitol at pH 9.81.

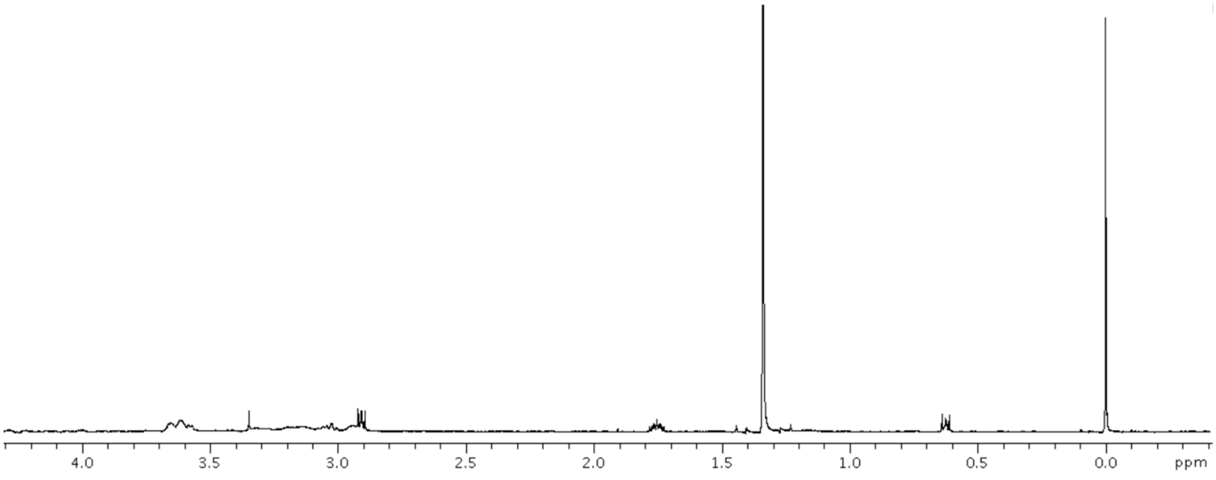

Figure S3. ^{1}H NMR spectrum of reaction mixture at equilibrium of selenocystine with dithiothreitol at pH 9.86.

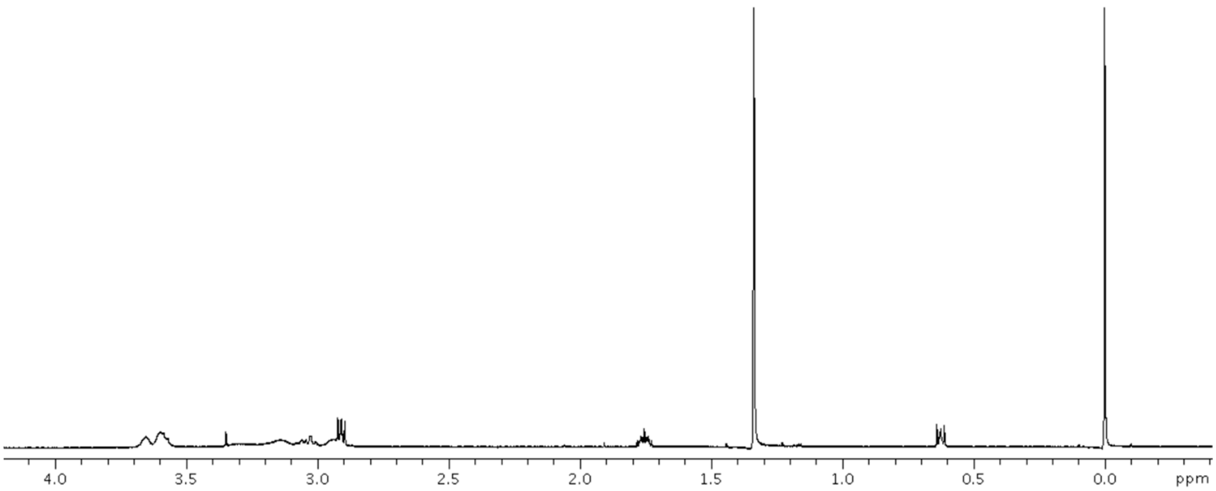

Figure S4. ^{1}H NMR spectrum of reaction mixture at equilibrium of selenocystine with dithiothreitol at pH 9.90.

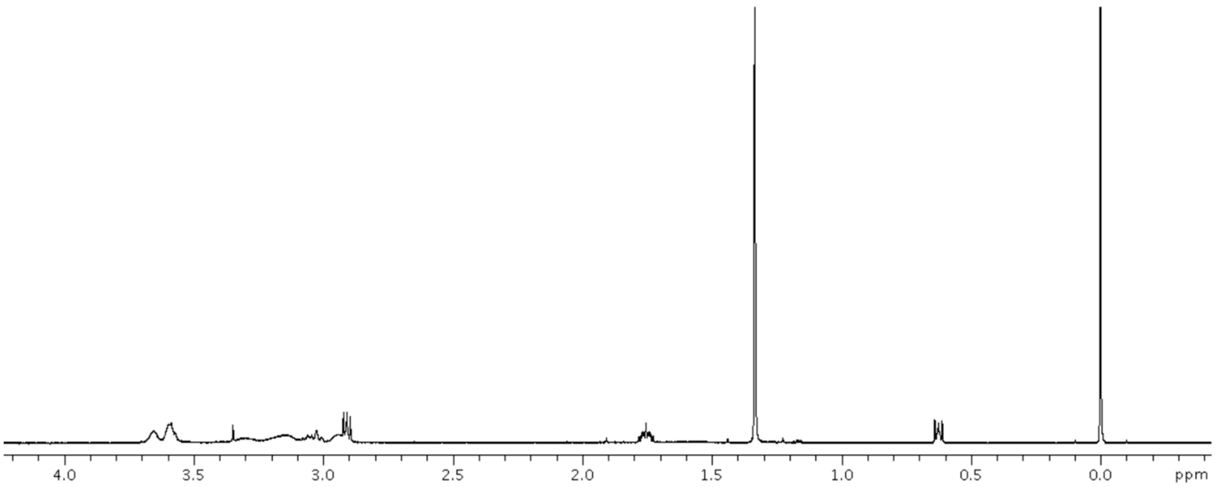

Figure S5. ^{1}H NMR spectrum of reaction mixture at equilibrium of selenocystine with dithiothreitol at pH 9.96.

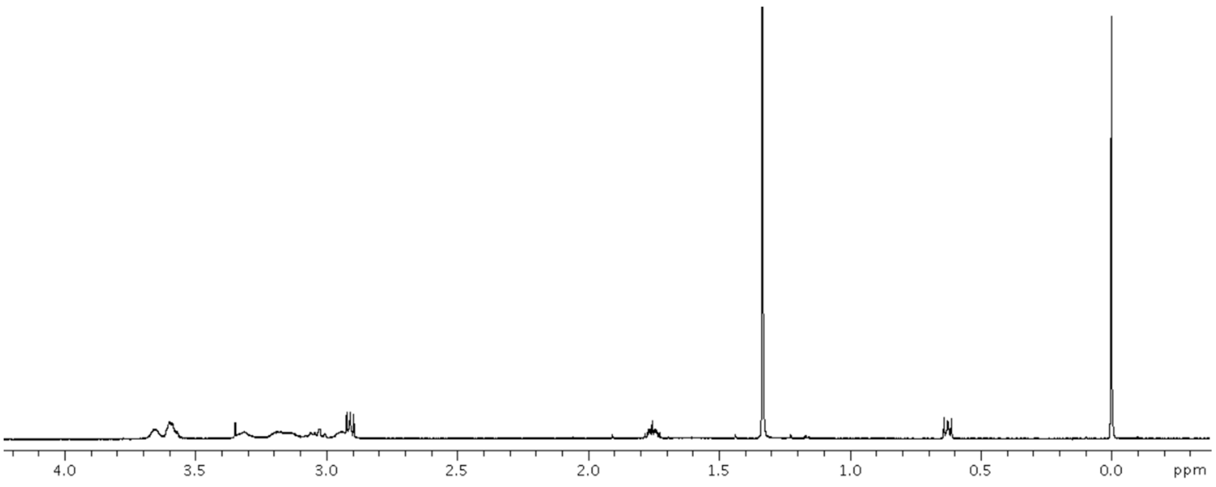

Figure S6. ^{1}H NMR spectrum of reaction mixture at equilibrium of selenocystine with dithiothreitol at pH 9.98.

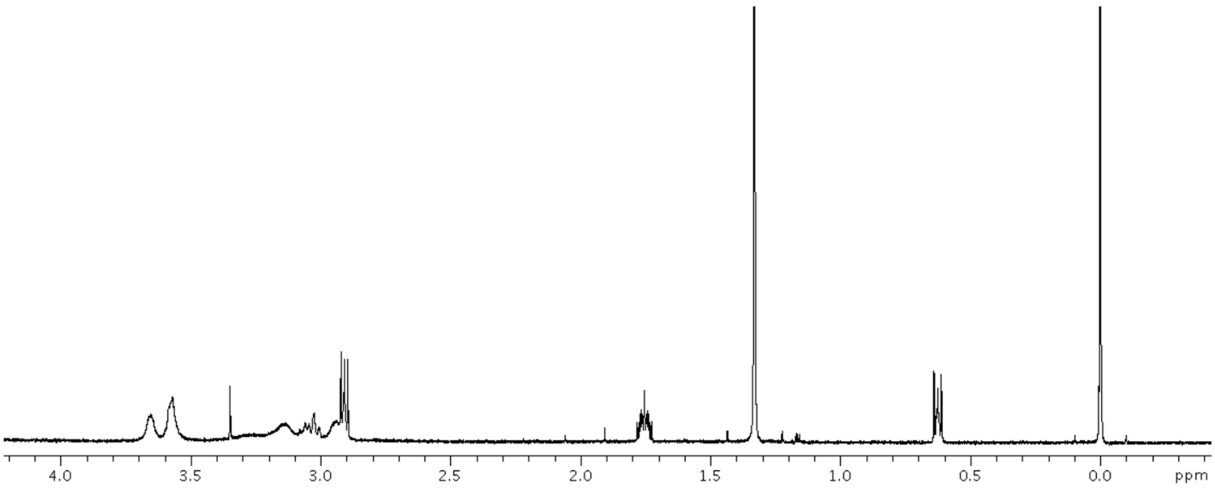

Figure S7. ^{1}H NMR spectrum of reaction mixture at equilibrium of selenocystine with dithiothreitol at pH 10.02.

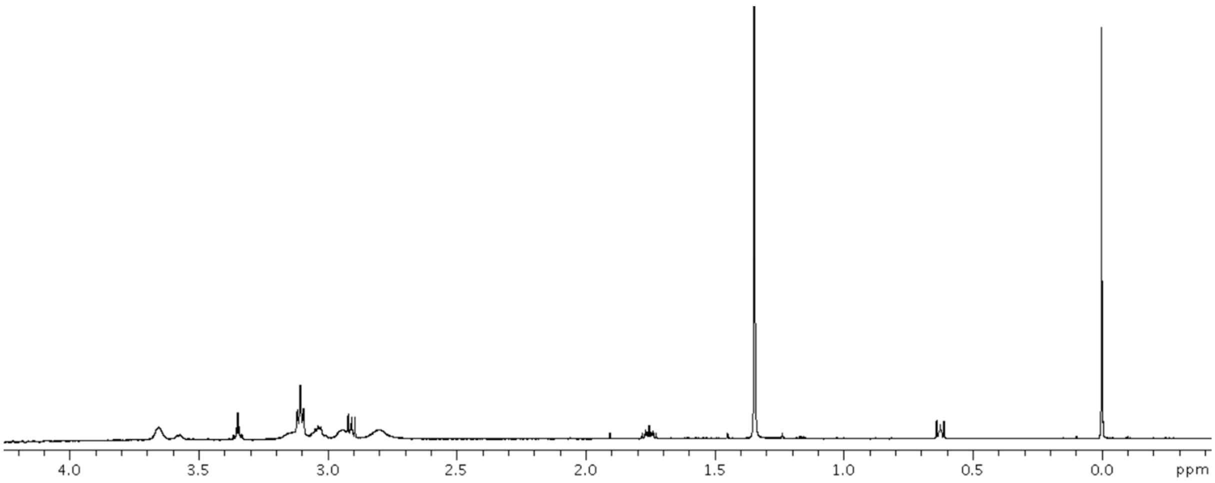

Figure S8. ^{1}H NMR spectrum of reaction mixture at equilibrium of selenocystamine with dithiothreitol at pH 9.66.

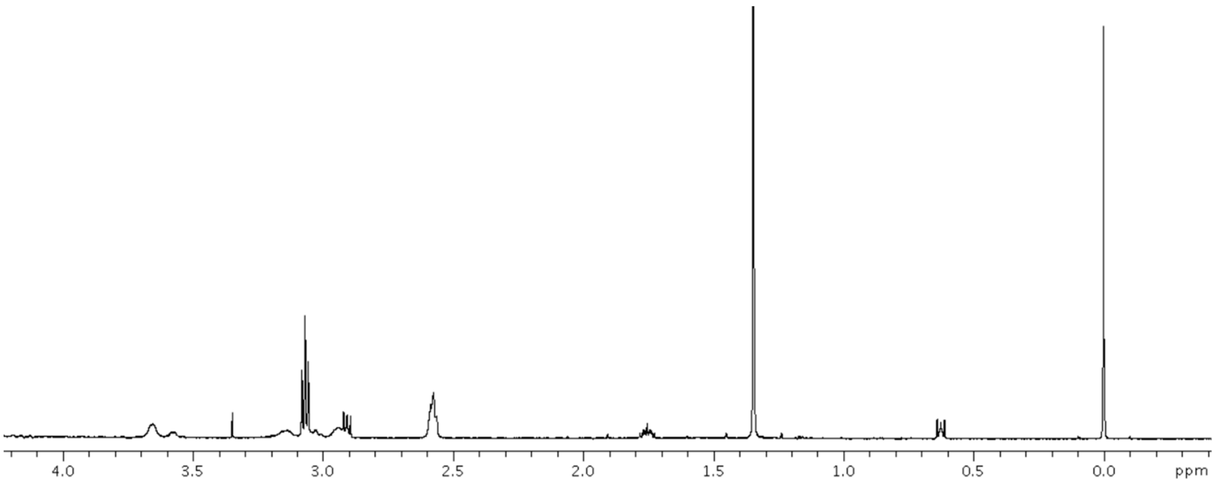

Figure S9. ^{1}H NMR spectrum of reaction mixture at equilibrium of selenocystamine with dithiothreitol at pH 9.67(a).

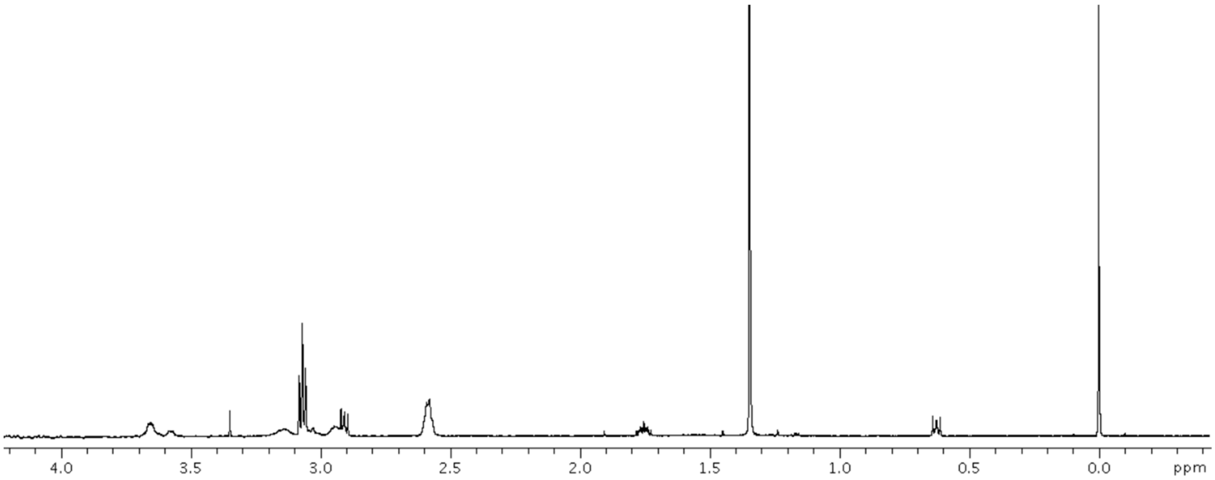

Figure S10. ^{1}H NMR spectrum of reaction mixture at equilibrium of selenocystamine with dithiothreitol at pH 9.67(b).

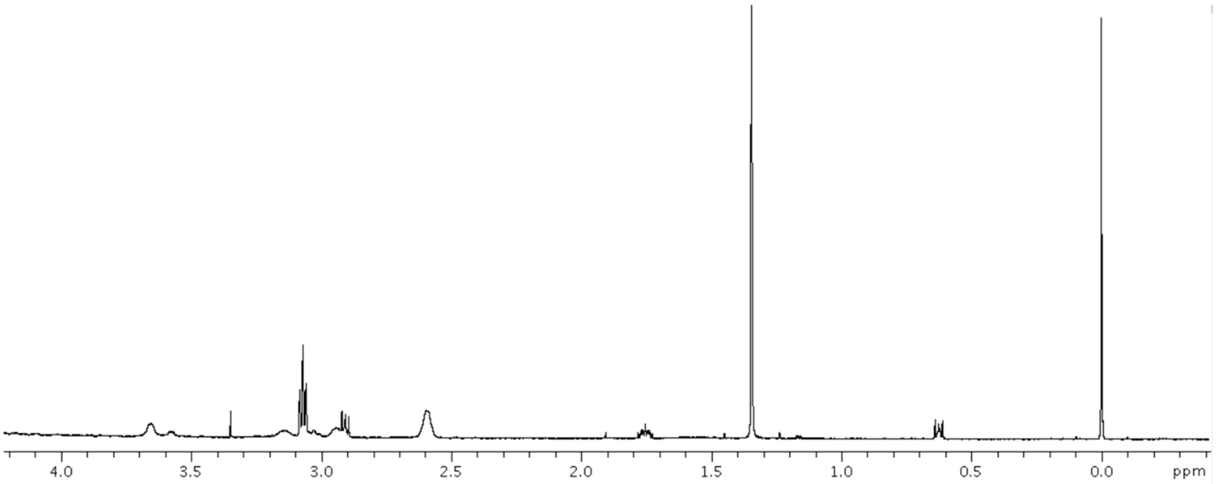

Figure S11. ^{1}H NMR spectrum of reaction mixture at equilibrium of selenocystamine with dithiothreitol at pH 9.68.

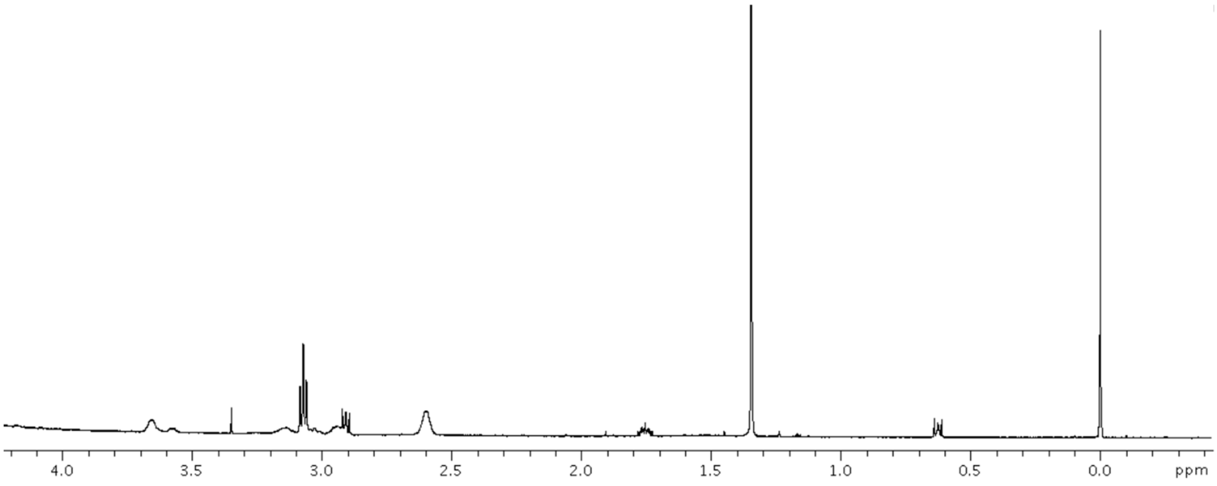

Figure S12. ^{1}H NMR spectrum of reaction mixture at equilibrium of selenocystamine with dithiothreitol at pH 9.69.

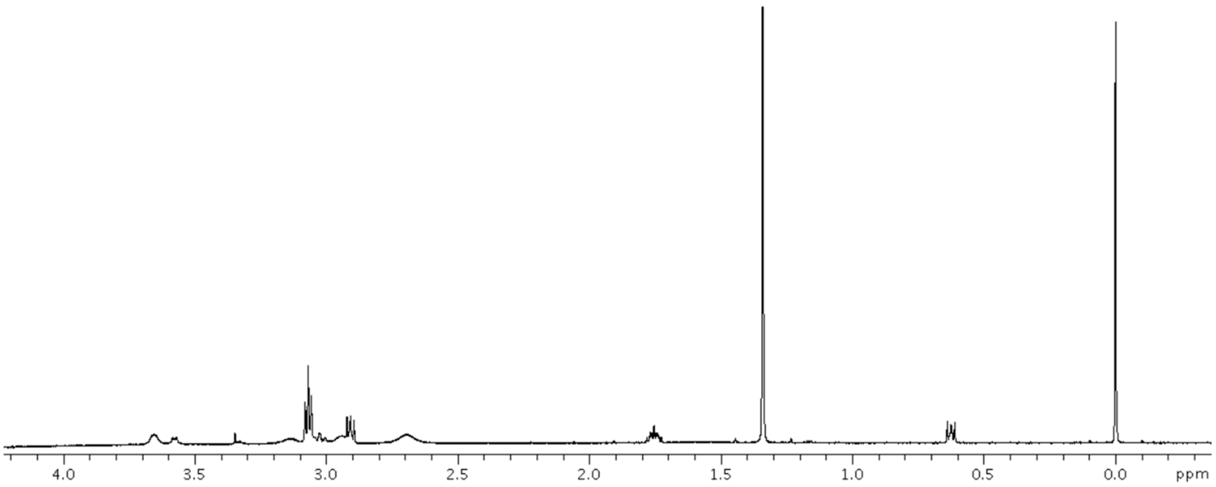

Figure S13. ^{1}H NMR spectrum of reaction mixture at equilibrium of selenocystamine with dithiothreitol at pH 9.80.

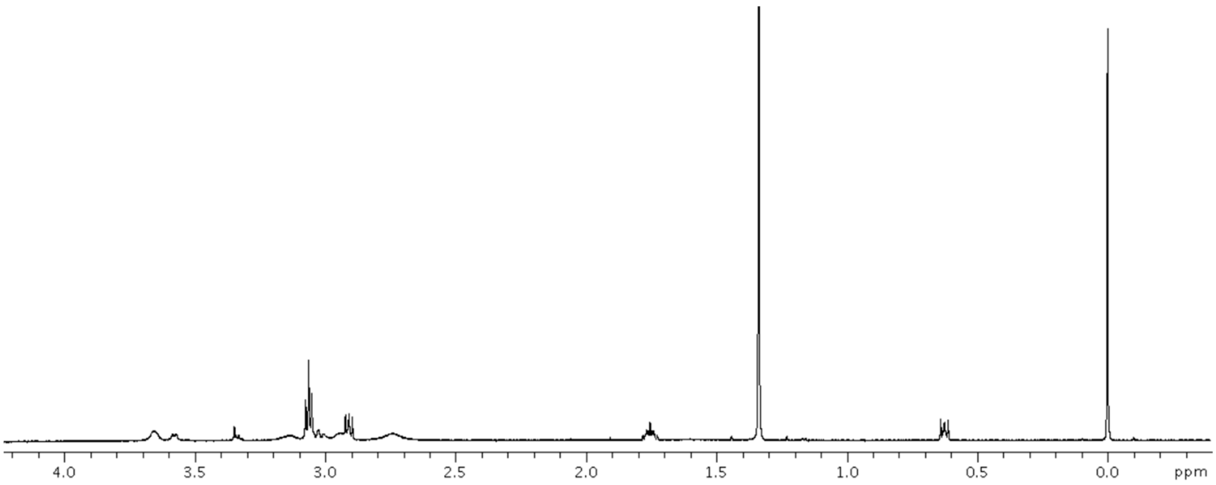

Figure S14. ^{1}H NMR spectrum of reaction mixture at equilibrium of selenocystamine with dithiothreitol at pH 9.89.

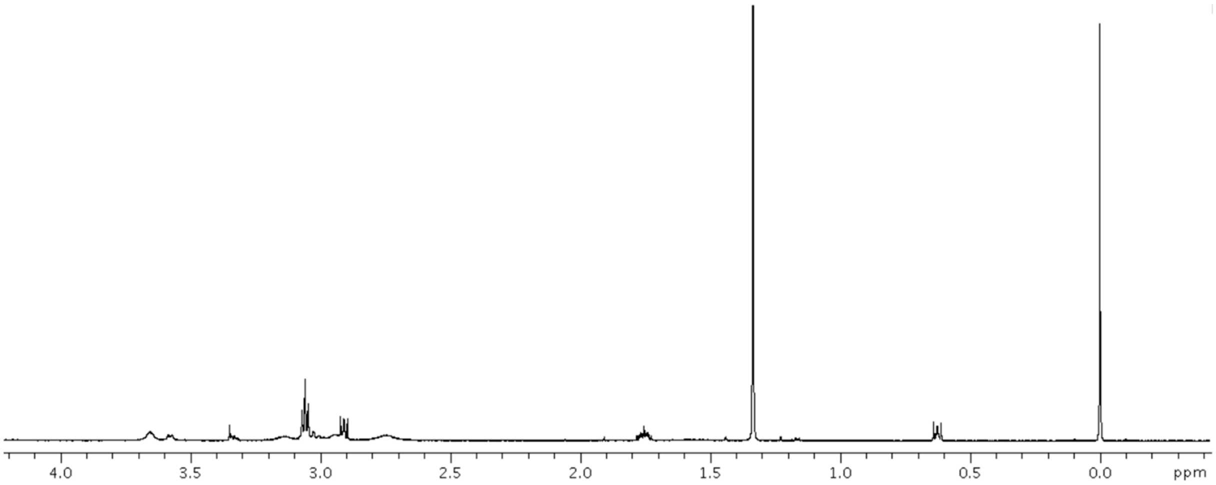

Figure S15. ^{1}H NMR spectrum of reaction mixture at equilibrium of selenocystamine with dithiothreitol at pH 9.94.

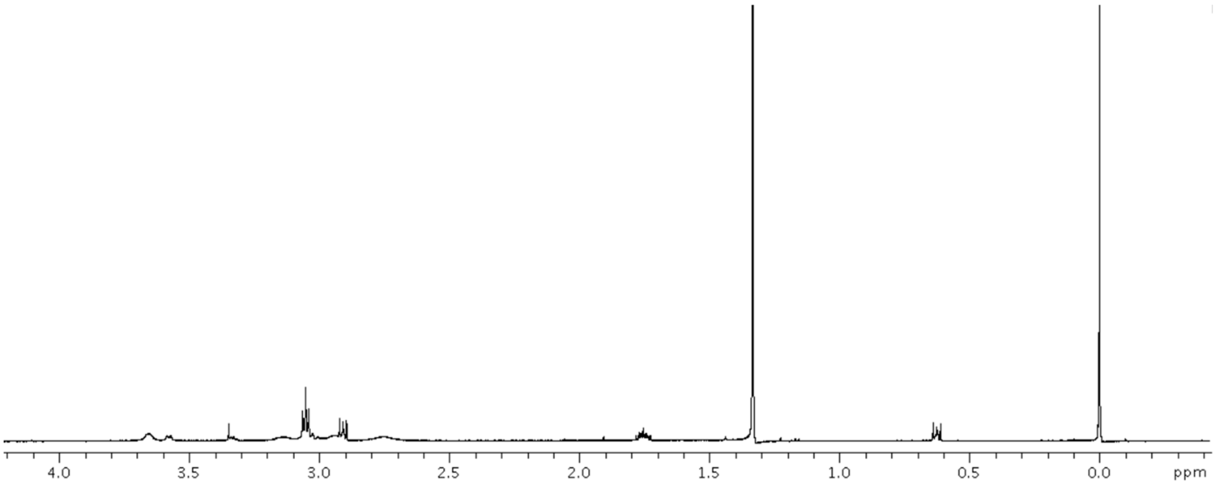

Figure S16. ^{1}H NMR spectrum of reaction mixture at equilibrium of selenocysteamine with dithiothreitol at pH 9.98.

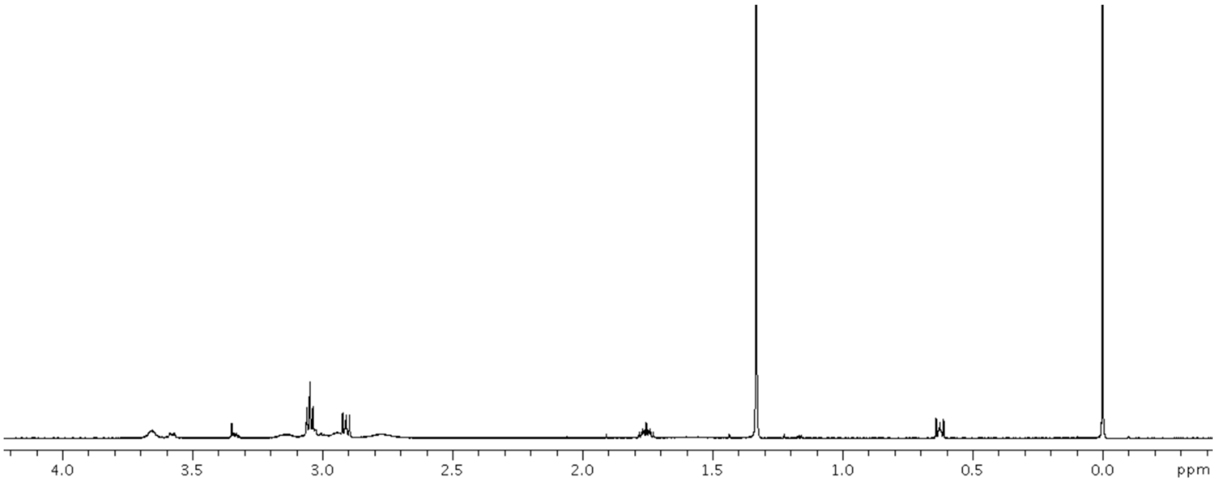

Figure S17. ^{1}H NMR spectrum of reaction mixture at equilibrium of selenocystamine with dithiothreitol at pH 10.01.

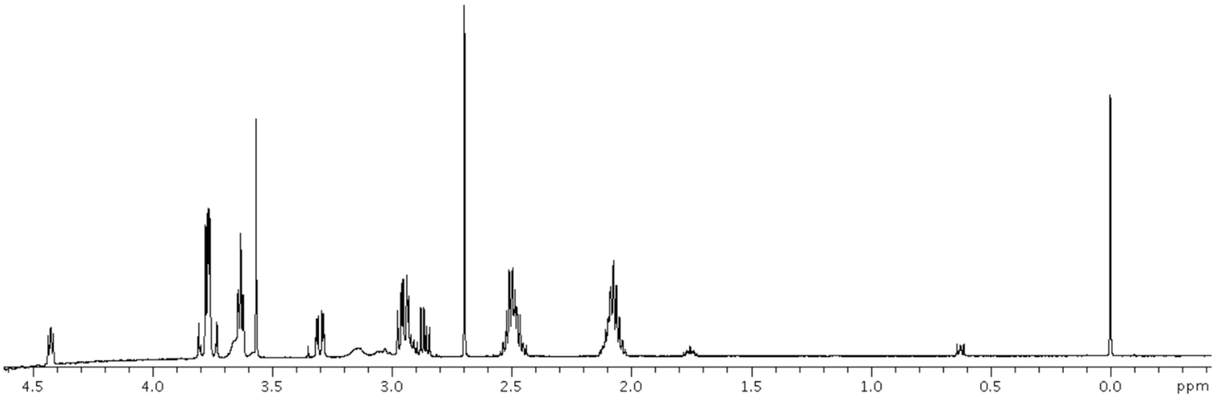

Figure S18. ^{1}H NMR spectrum of reaction mixture at equilibrium of oxidized glutathione with dithiothreitol at pH 9.09.

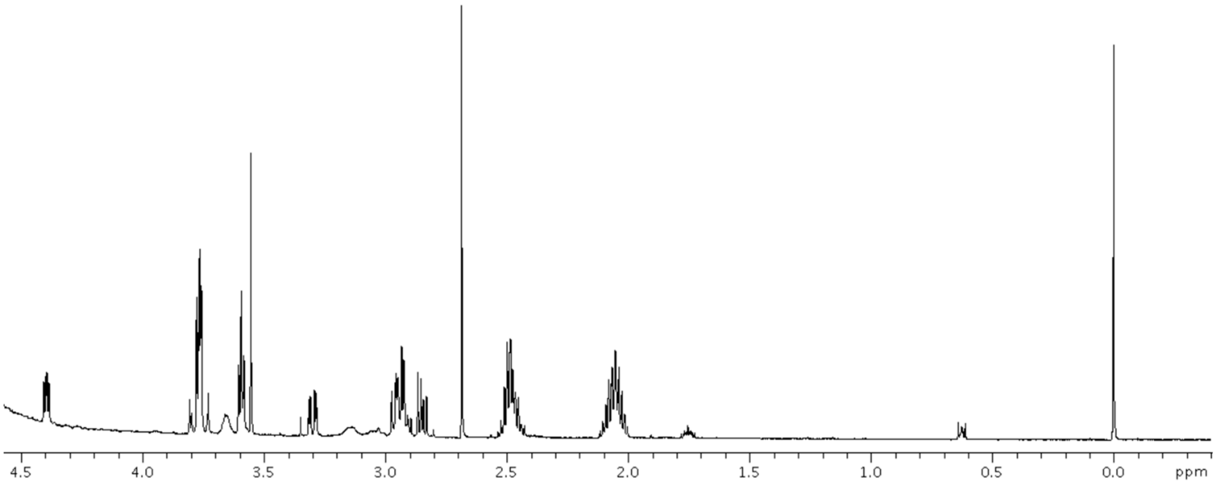

Figure S19. ^{1}H NMR spectrum of reaction mixture at equilibrium of oxidized glutathione with dithiothreitol at pH 9.23(a).

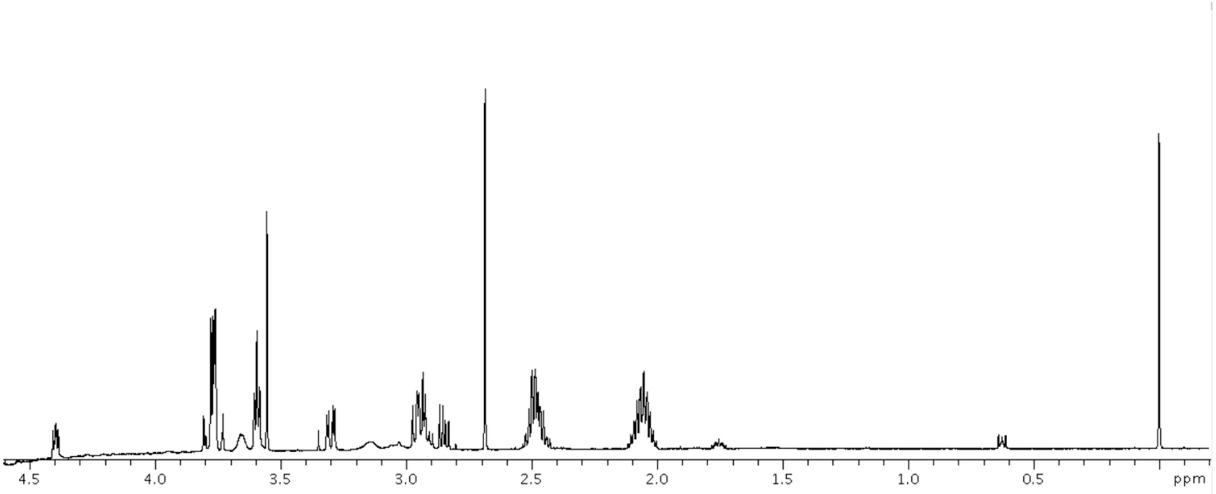

Figure S20. ^{1}H NMR spectrum of reaction mixture at equilibrium ofoxidized glutathione with dithiothreitol at pH 9.23(b).

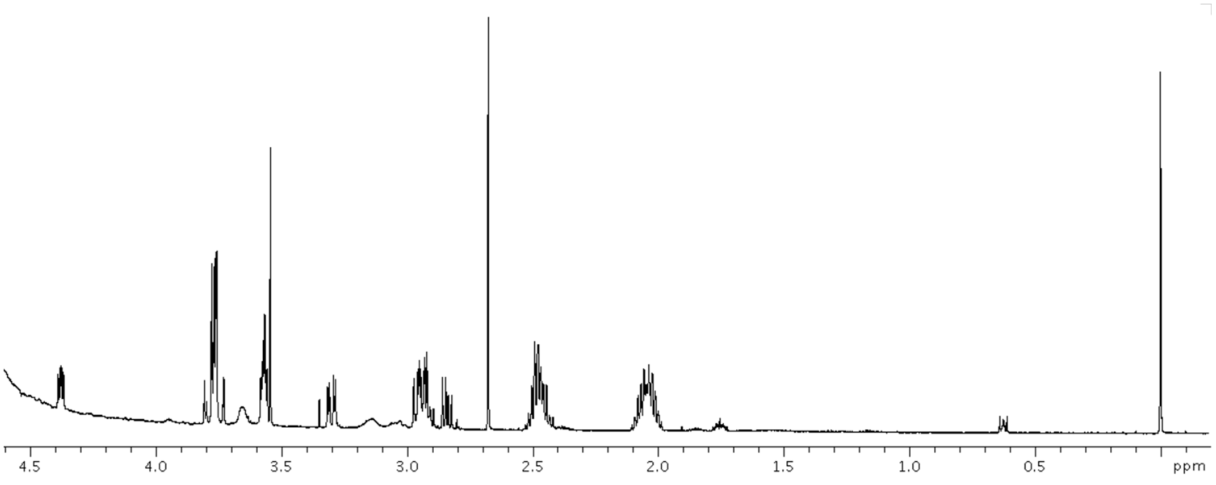

Figure S21. ^{1}H NMR spectrum of reaction mixture at equilibrium of oxidized glutathione with dithiothreitol at pH 9.32.

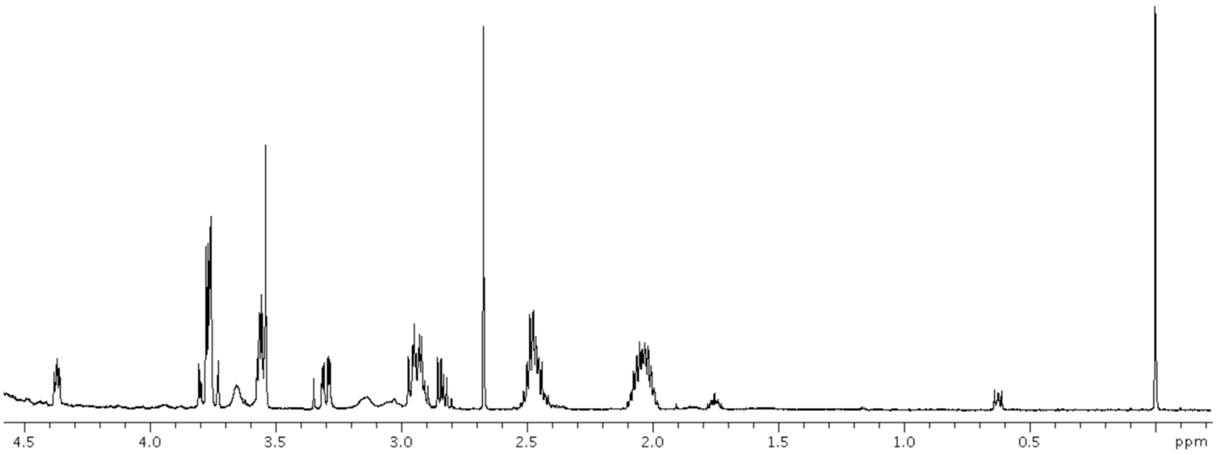

Figure S22. ^{1}H NMR spectrum of reaction mixture at equilibrium of oxidized glutathione with dithiothreitol at pH 9.35.

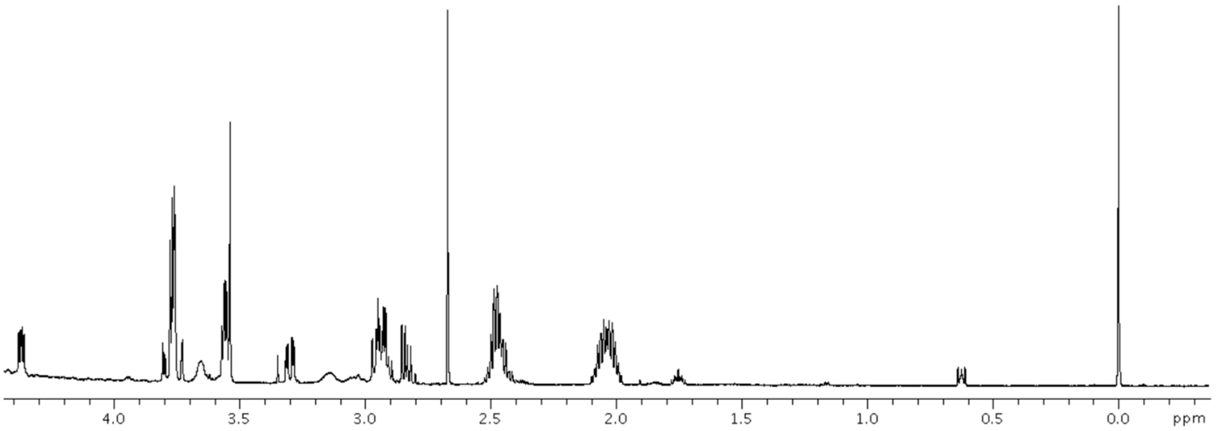

Figure S23. ^{1}H NMR spectrum of reaction mixture at equilibrium of oxidized glutathione with dithiothreitol at pH 9.37(a).

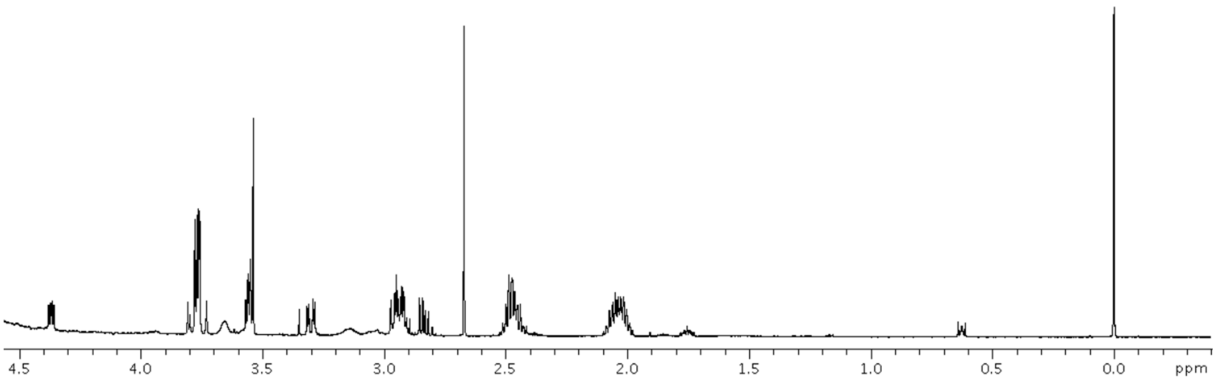

Figure S24. ^{1}H NMR spectrum of reaction mixture at equilibrium of oxidized glutathione with dithiothreitol at pH 9.37(b).

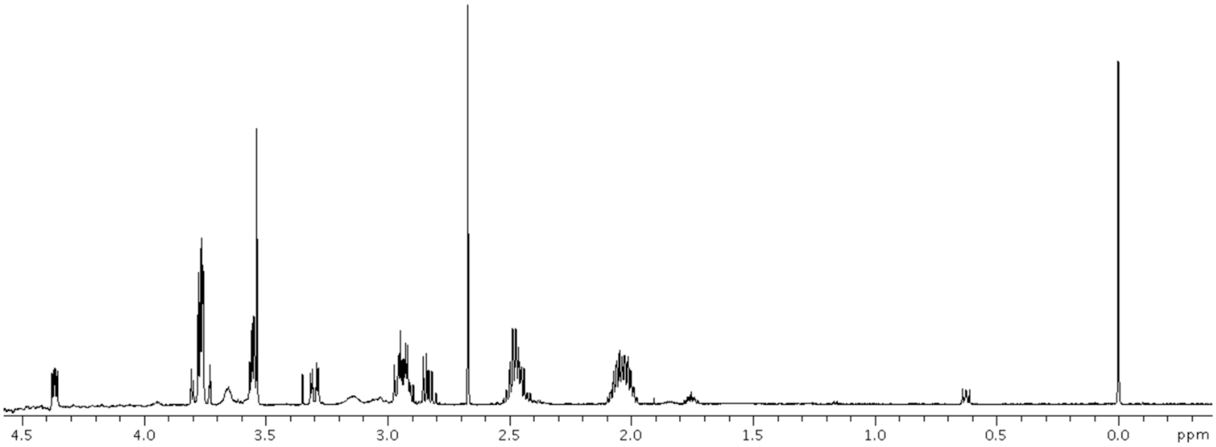

Figure S25. ^{1}H NMR spectrum of reaction mixture at equilibrium of oxidized glutathione with dithiothreitol at pH 9.38.

Table S1. The integral area of peaks and calculated concentrations of reaction mixture compounds.

				Selenocystine - DTT			
**pH**	**Area (reference)**	**Area (DTT_{ox})**	**c(reference) mol/L**	**c(CysSeSeCys) mol/L**	**c(CysSeH) mol/L**	**c(DTT) mol/L**	c(DTT_{ox}) mol/L
9.79	37673044	1447681	0.003275	0.000594	0.001133	0.000936	0.000566
9.86	38237500	1489676	0.003149	0.000563	0.001104	0.000892	0.000552
9.81	16199326	2129353	0.00126	0.000375	0.00149	0.000695	0.000745
9.90	15048319	2084890	0.00120	0.000318	0.001494	0.000623	0.000747
9.96	14644780	2101871	0.00116	0.000283	0.001502	0.000578	0.000751
9.98	14442950	2038241	0.00114	0.000289	0.001443	0.000577	0.000721
10.02	14725975	2078625	0.00111	0.000282	0.001410	0.000564	0.000705
				Selenocystamine - DTT			
pH	Area (reference)	Area (DTT_{ox})	c(reference) mol/L	c(CysASeSeCysA) mol/L	c(CysASeH) mol/L	c(DTT) mol/L	c(DTT_{ox}) mol/L
9.80	19010492	1950064	0.00148	0.000394	0.001366	0.000766	0.000683
9.89	17680668	2039621	0.001407	0.000294	0.001461	0.000648	0.000731
9.94	17337042	1893230	0.001366	0.000323	0.001342	0.000667	0.000671
9.98	16895478	1880989	0.001334	0.000303	0.001337	0.000638	0.000668
10.01	16356080	1861289	0.001304	0.000281	0.001336	0.00061	0.000668
9.66	145359264	18788642	0.00124	0.000424	0.001442	0.000719	0.000721
9.67	132270976	16435223	0.001179	0.00043	0.001319	0.00071	0.000659
9.67	134577520	18201112	0.001144	0.000361	0.001393	0.000633	0.000696
9.68	130598720	16043556	0.001118	0.000415	0.001236	0.00068	0.000618
9.69	127561664	16220745	0.001093	0.000384	0.00125	0.000644	0.000625
				Oxidized glutathione - DTT			
pH	Area (reference)	Area (DTT_{ox})	c(reference) mol/L	c(GSSG) mol/L	c(GSH) mol/L	c(DTT) mol/L	c(DTT_{ox}) mol/L
9.23	32601690	22598261	0.001706	0.002742	0.002364	0.000872	0.001182
9.37	34210608	22469643	0.001439	0.002366	0.001891	0.000788	0.000945
9.38	35613496	18370429	0.001399	0.002497	0.001443	0.000964	0.000722
9.09	46954896	33211823	0.00204	0.003247	0.002883	0.001013	0.001441
9.23	70377528	54048592	0.00171	0.002614	0.002620	0.000745	0.00131
9.32	30375060	16860640	0.00152	0.002651	0.001686	0.000986	0.000843
9.35	33984756	21639255	0.00144	0.002395	0.001833	0.000817	0.000916
9.37	35085112	18479023	0.00140	0.002482	0.001474	0.000948	0.000737
